# Sports activities of 60 above Hungarian elderly-explaining and predicting impact of exercise on health

**DOI:** 10.1186/s12889-020-09974-x

**Published:** 2021-04-23

**Authors:** Kinga Lampek, László Csóka, Réka Hegedüs, Miklós Zrínyi, Mária Törőcsik

**Affiliations:** 1 Department of Health Promotion and Public Health, Institute of Health Insurance, Faculty of Health Sciences, University of Pecs, Pécs, Hungary; 2 Department of Marketing and Tourism, Faculty of Business and Economics, University of Pecs, Pécs, Hungary; 3 Faculty of Health Sciences, University of Pecs, Pécs, Hungary

**Keywords:** Ageing, Sport, Old-age physical activity, Hungary

## Abstract

**Background:**

The proportion of elderly is on the rise both in Europe and in Hungary. The challenge is to increase the number of years spent in good health as well as to improve quality of life of those 60 years and above. This study focuses on the impact of physical activity on this age group.

**Methods:**

A nationally representative sample of 2000 respondents were surveyed in an age range of 15–74. Our data concerns those being 60–74 years of age. The focus of our investigation was level and impact of physical activity in the group above. First, we used Chi-squared tests and correspondence analysis to identify the deviation in the answers of different groups in our sample. After we built a hierarchical linear regression model to get a deeper understanding of the impact of physical activity for elderly.

**Results:**

Those reporting no physical/sports activity at all have to do with the negative culture of exercising. Only 9.3% reported being engaged with any sports; 72% reported no regular exercising throughout their lives. The relationship between sport activity and self-reported health was significant (*p* = 0.009, Cramer’s V = 0.2). Elderly were characterized by walking, hiking and less intense sports.

**Conclusions:**

Those who actively exercised in this research reported better health outcomes than those who stopped or had never been engaged in any sports. We conclude that of all variables tested, physical activity was most effective to improve personal health of the elderly in this sample. Compared to European data on physical activity of elderly populations, Hungary seems to fall behind and needs to consider concentrated efforts to improve the future health of its senior populace.

## Background

The number of ageing people has been on the rise in the last decade. In the year 2017 a total of 962 million, aged 60 or above individuals have been recorded which is about 13% of the population of the world. The ratio of elderly is greatest in Europe (25%). By estimation, the world will have about 3.1 billion old persons in 2100 [1].

In order to describe where old age begins there seems to be a misperception in the literature. To define young, middle and old age generations, a gap has emerged that makes it more difficult to find a solid cut point [2]. In this paper, old age has been defined as 60 years and above, reason being that due to various sociopolitical regulations some members of society are able to retire at age 60, hence inactivity, the main interest of our inquiry, may begin. As of 2019, the National Bureau of Statistics reported 2.6 million people living 60 or above, that is 27.1% of the total population of Hungary. Of those in this age group 60% were women, 13.7% were reported to be 60–69 years old, 8.8% between 70 and 79 of age, and 4.6% above 80 years [3]. Their proportion will continue to increase, people 80 years or above is expected to reach 29% by 2070 as forecasted by Eurostat [4–6]. Life expectancy at birth in the countries of the EU was 83.3 years for women and 77.9 for men while Hungary reported 79.0 for women and 72.4 for men [7].

There is a complex need to intervene in order to increase the life expectancy as well as quality of life of senior citizens. One area of improvement clearly is physical activity. A nationally representative sample of 2000 respondents were surveyed in May–June of 2018. The survey was implemented in an age range of 15–74, our data concerns those who reported their age being 60–74. The focus of our investigation was the level of physical and sports activity in the group above. Our hypotheses included that doing sports at older age is positively related to life satisfaction as well as not being engaged in physical activity has to do with earlier socialization that lacked exercising culture. We aimed to explore whether elderly physical activity, namely types of sports, are different from other generations.

International literature describes physical activities and activities of daily living concerning the elderly. Literature is less replete with studies exploring and evaluating sports acitivies of older people, there seems to be a specific lack of studies about the motivation of elderly to partcipate in sports and why they withdraw from sports. However, such studies would help us understand how to engage elderly generations and ensure that they enjoy health benefits of sporting. In this particular study, we made an effort to quantify the influence of sport both on quality of life and self-assessed health. Our study outcomes further confirm that the level of elderly engagement in sports activities is unnecessarily low.

### Impact of physical activity on health

Physical activity has been found to have a string positive impact on health outcomes, especially in prevention of chronic diseases and early death. Exercising positively impacts mood, boosts self-efficacy and improves coping with stress. Regular physical activity has been observed to improve social relations, cohesion and also social support [8–13]. In older age, physical activity has a positive influence on quality of life and mental health of individuals [14, 15]. It is very effective in maintaining physical balance and preventing falls [16, 17]. Physical exercise may also help to reduce the cost of healthcare by preventing or pushing out disease onset to a later age [18]. Epidemiological studies prove that physical inactivity is the biggest factor of mortality overtaking smoking and high blood pressure [19]. Lack of exercise is said to cause at least 5 million death annually in the world as it may be connected to 6% of cardiovascular diseases, to 7% of type 2 diabetes, to 10% of breast and colon cancers, and is a known factor of osteoporosis and precursor of depression [20, 21]. The level, intensity and frequency of exercise for a balanced and healthy life has been defined by the World Health Organization, the European Council and the US government as well [22–24]. These guidelines include very specific recommendations for daily activities for those 65 years old and above to enjoy full mobility, independent functioning and mental health.

### Impact of physical activity on health in Hungary

Old age in Hungary is often associated with decline, elderly respondents believe that it is difficult to adjust to age related challenges, they become dependent on others, their physical and mental health deteriorated, altogether negative associations are predominant [5, 25]. According to the Global Age Watch Index in 2015 they found that out of the 96 countries surveyed Hungary ranked 57th based on health conditions and well-being of the elderly [26]. Health status of people 65 and above in Hungary was appraised in 2016 explicitly unsatisfactory by 18% of respondents, 10% said they were quite disappointed with their health, about a quarter reported being satisfied [27, 28]. When considering genders, women are more critical of their health status than men. The process of aging brings forward chronic diseases that negatively impacts on mobility and exercise [29]. Chronic diseases affect 80% of the elderly population, 39% report serious restrictions of mobility and sensory disabilities while 35% are affected by diseases that prevent them from full functioning and managing a household [5]. Most prevalent diseases in this population include high blood pressure (66.9%), back and lumbar pain (51.4%), joint diseases (46.8%), neck pain (27.5%), cardiac fibrillation (23.4%), high cholesterol (22.9%) and osteoporosis (21.1%). Based on the European Household Survey 2014 data one in seven Hungarian is engaged in physical activity, 5% exercises daily, 2% weekly on 1–3 occasions. While 25% of this sample reported biking another 25% did not walk more than 10 min weekly. Despite that doing sports has been proven to reduce cardiovascular morbidity and contributes to better weight management [30–33], physical inactivity has been well documented in Hungarian literature [18, 34–36]. Exercise activity increases by level of education, the more people are educated the more affluent they become thus they spend time and resources on sports more than lower educated peers [5, 37]. Regularly exercising men, those living in cities and having higher education, reported better health compared to peers [38–40]. Retirement however causes people to change lifestyles, restrict their activities in and around their homes, and recreation activities are pushed back significantly. Loss of certain muscular functions and independent living reduce physical activity that may increase the risk of cardiovascular disease [41, 42]. Regular exercise however can minimize the risk of osteoporosis and diseases that affect the locomotive system [43]. Fear from falling may also decrease physical activity in 65 and older, research shows that at least one-third of people at and above the age of 65 fall annually [44]. Reasons behind falls are numerous; instability, muscle tone changes, weight changes and the slowing of the nervous system may all contribute to losing stature. The loss of physical activity can easily lead to depressive symptoms which then further exacerbates loneliness and a worsening quality of life [45]. Exercise is a critical tool to keep the elderly socially integrated and to avoid serious disabilities. Health promotion including improving exercise capacity is not late beyond 60 years of age as such programs have proven to be effective in maintaining independent living and functioning [46–48].

As for the comparison with other European countries, Eurobarometer 2017 data showed marked differences in the motivation of elderly between East and West. Elderly in Western Europe find it important to exercise in order to stay fit and healthy, be in control over their weight, and use exercise as means of relaxation and social bonding. Clearly, while 49.2% of 60-above in Western Europe walked at least 10 min 5 days a week, the proportion of the same age group was only 33.1% in Hungary.

### Measuring physical activity

According to Ainsworth, physical activity may be measured by physical assessments (i.e., gait and velocity meters, heart rate monitors etc.) or other quantitative/qualitative tools such as personal interviews and surveys [49, 50]. For greater number of subjects however using survey tools is more frequent as they can collect a large amount of data in a relatively standardized, comparable fashion. There are a number of validated questionnaires available on the topic of physical activity that have been tested in large population surveys. Among those, the European Activity Surveillance System (EUPASS) is the most dominant in Europe [51]. Another tool, by recommendation of WHO, used in Europe is the International Physical Activity Questionnaire (IPAQ) [52, 53] and its revised sibling the Global Physical Activity Questionnaire [54, 55]. As for measuring sports activities across Europe, a valid approach has been the Eurobarometer study in 2002, 2009, 2013 and 2017 [56–58]. The last survey concerned 28 European member states and included data from 28,031 European citizens from various sociodemographic groups. The survey assessed the level and frequency of physical activity from moderate to intense and accounted for activities like walking. It also evaluated the place and venue of the exercise including riding a bike, dancing or gardening. The survey explored reasons why people chose or declined to be active and reasons that either supported or impeded being physically active including the role of municipal governments and local environments. Finally, the survey also looked at whether people took any voluntary role in supporting sport events [34]. The final instrument utilized in this research reflected items and measurement principles both of EUPASS and IPAQ. Additional survey items were added to the instrument in order to account for cultural values and differences.

## Methods

A nationally representative survey on a panel of 2000 respondents between May–June 2018 was implemented. Subjects were identified through the national register of citizens and were contacted over the phone to sit for the interview. All interviews were conducted by a trained interviewer in respondents’ homes. The average time of an interview took 30 to 40 min. The age range of the panel included 15–74-year-old subjects. The sample was representative and proportionate of age (10 years intervals), gender and region. The selection of the household followed the so-called ‘random area walk-in’ technique, while the actual interviewee was selected by using the last birthday digits. Using a 13-page instrument, respondents were surveyed on their appraisal of exercise activity and sports as well as consumption of sports related goods and services. The instrument was developed based on learnings from EUPASS and IPAQ, however, validity of the instrument was not established in this research. None of the items were adapted directly from EUPASS and IPAQ. Majority of the final items were developed by a panel of 5 experts in the field who ensured face validity for the final instrument. One exception was the instrument for ‘lifestyle-inspiration’ where the full scale was implemented from the original. The scale has been validated earlier [59]. Reliability for individual dimensions of the scale were as follow: 0.888, 0.885, 0.887, 0.896. While reliability is not guarantee for validity, however, lack of validity is usually accompanied by low reliability. Based on above reliability values we therefore assume that the instrument had substantial validity besides the face validity established by the expert panel.

Responses were quantitative and analyzed for the assessment of the hypotheses below:

- There is a significant, positive relationship between elderly exercise and satisfaction with life
- Non active elderly lifestyle is associated with the lack of exercise culture
- Sport engagements of the elderly significantly differ from those of younger generations

Chi-square analysis was used to answer to hypotheses 1–2 where distinct age groups have been determined to investigate the relationships stated above. To assess hypothesis 3, correspondence analysis was applied. Outside hypotheses, we decided to build a hierarchical regression model to predict appraisal of personal health by lifestyle, exercise, BMI and satisfaction with life variables. Hierarchical regression was implemented to predict self-reported health outcomes of respondents by using life satisfaction, BMI, and other factors. Outliers had been identified as having a standardized residual ±2.0 and were removed from the analysis. All analyses were performed level of significance set at 5%. To run the analyses IBM SPSS Statistics version 25 was used. There was no specific policy developed to handle missing data, such data were excluded from further analyses.

To investigate age related (generational) differences, respondents were classified into individual groups. In order to separate respondents into distinct groups, several choices were available: e.g. classic cohorts [60, 61] and media driven generational classification [62]. Besides the ‘old’ group cannot be considered as a homogeneous category (‘young old’ between 65 and 74, ‘old’ between 75 and 84, and ‘oldest old’ over 85 years of age) but for the purposes of this paper, in order to arrange an appropriate number of older participants in the sample, we chose the classic cohort classification, namely 30 years and below were grouped as ‘young’, those between 30 and 59 years of age as ‘middle generation’ and 60 years and above were categorized as ‘elderly’.

A priori sample size estimation (using GPower version 3.1.9.3) for hierarchical regression (significance set at 5%, power set at 0.8, effects size at 0.15, and number of predictors at 8) showed that a total of 109 subjects were required to ensure adequate statistical power for analyses. The final sample of 464 subjects exceeded sample requirements.

## Results

**Fig. 1:**
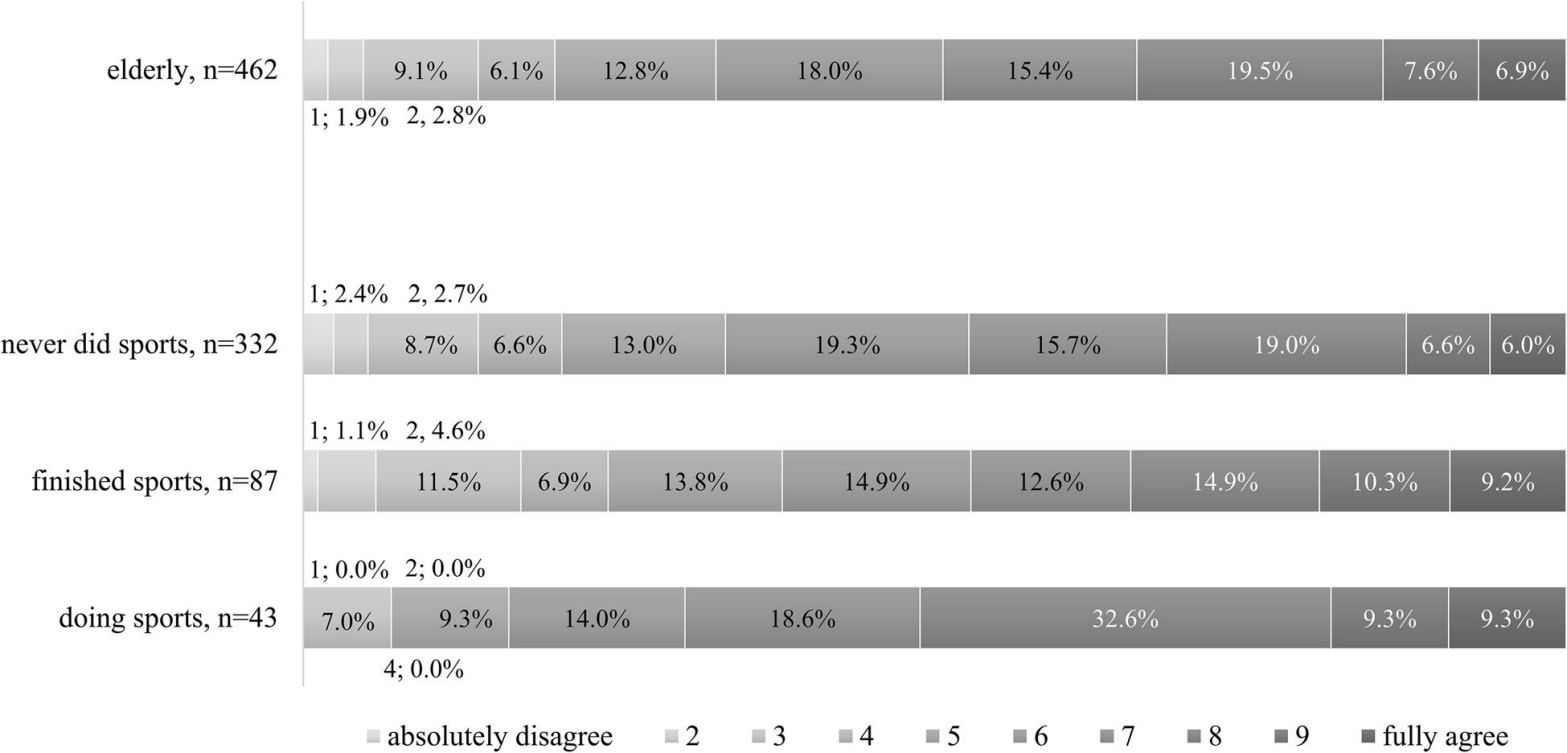
Satisfaction with life by sport activity for the elderly (*p* = 0.50). “How satisfied are you with your life?”

Sample characteristics and descriptive statistics are presented in Table 1. To assess satisfaction with life, a 10-point Likert scale was used where 1 denoted ‘absolutely not satisfied’ and 10 indicated ‘absolutely satisfied’. Results showed a significant difference in life satisfaction across generations. For the 10-point Likert scale we considered answers from 1 to 4 points as the disagree side of the scale and answers 7 to 10 points as the agree side of the scale. Answers from 5 to 6 points are considered neutral. The most satisfied generation was the young population, they scored in 66.6% 7 or greater on the satisfaction scale. Those in the older generation were the least satisfied they scored 7 or above on the scale less frequently (35.5%) compared to others. When life satisfaction was contrasted with sport activities, a medium strengths relationship was observed (Cramer’s V = 0.197; *p* = 0.000). Those who were most satisfied with their life (scores above 7.0 68.2% of the time) had actively been engaged in sports activities as well (Fig. 1). Those who stopped working out (scores above 7.0 46.6%) or never worked out (scores above 7.0 42.2%) were less satisfied. One conclusion of this paper therefore was that actively exercising improved the sense of well-being and satisfaction in our elderly sample.

**Table 1:** Demographic data of the old generation (*n* = 464)

	Capita	%		Capita	%
Gender			Marital status		
male	214	46,1%	single	17	3,6%
female	251	53,9%	married	250	53,8%
Highest level of education			divorced	69	14,9%
Primary (up to 8 years of elementary school)	278	59,8%	widowed	110	23,8%
Secondary level (vocational training, vocational secondary school, high school)	120	25,9%	registered partnership	18	3,9%
Higher education (advanced technical school, bachelor’s degree, master’s degree)	65	14,1%	Region		
no respond	1	0,2%	Central Hungary	130	28,1%
Economic activity			Central Transdanubia	53	11,5%
Active blue-collar worker	50	10,8%	West Pannon	46	9,9%
Active white-collar worker	29	6,2%	South Transdanubia	43	9,2%
Retired	373	80,3%	Northern Hungary	60	13,0%
Unemployed	3	0,7%	Northern Great Plain	71	15,3%
Other inactive status	7	1,5%	Southern Great Plain	61	13,0%
no respond	2	0,4%	Settlement		
Monthly income level			Budapest	79	17,0%
We make a living by it very well and we can spare money.	141	30,3%	city with county rights	99	21,2%
Just enough to make a living by it, and we can’t spare.	256	55,2%	city	138	29,7%
We have living problems regularly.	51	11,0%	village	149	32,1%
no respond	16	3,5%	Number of the regularly athletes in the household		
The number of people living in the same household			0	392	84,4%
1	160	34,4%	1	52	11,2%
2	238	51,1%	2	14	3,0%
3	36	7,8%	3	6	1,2%
4	16	3,4%	4	1	0,2%
5	6	1,3%	State of health limits sport activity		
6	6	1,3%	yes	236	50,9%
7	1	0,2%	no	220	47,4%
no respond	2	0,5%	no respond	8	1,7%

**Fig. 2:**
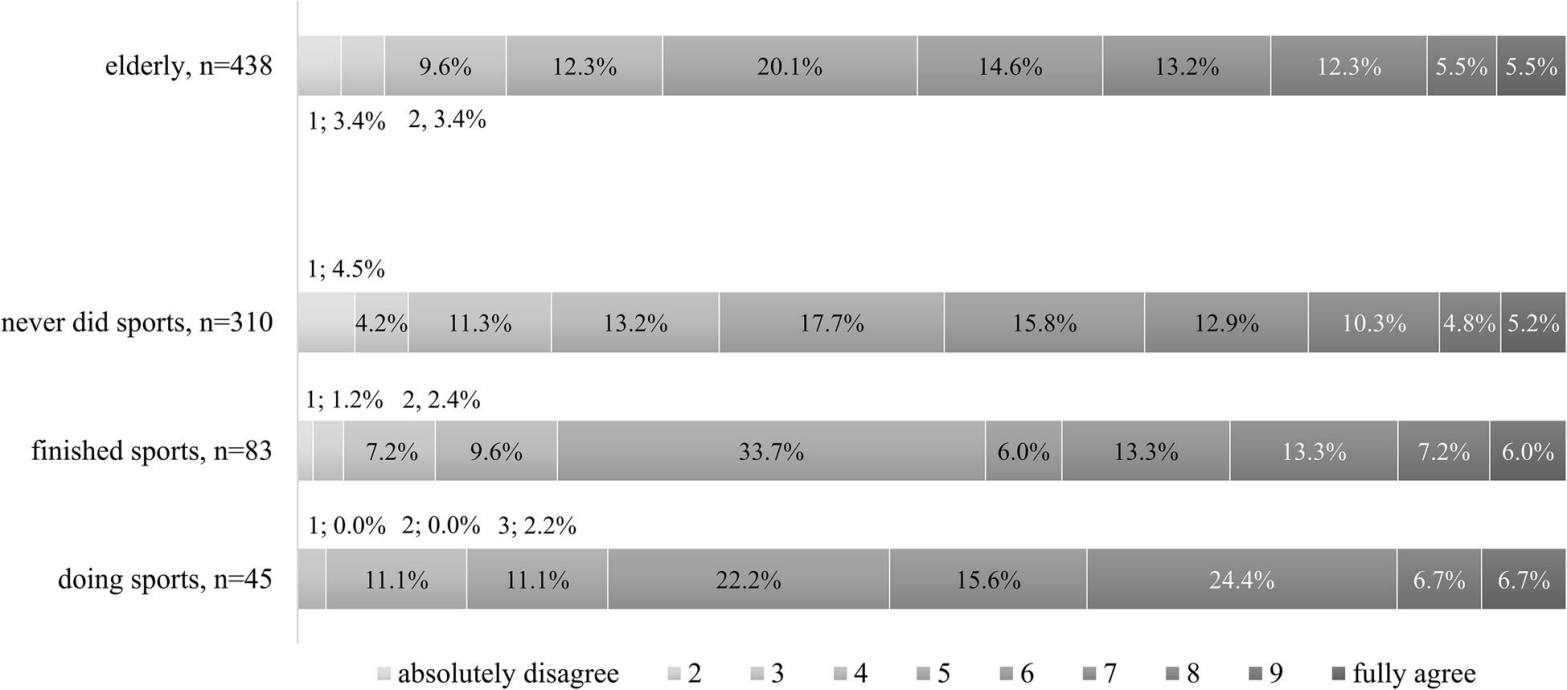
Association between health and sport activities for the elderly (*p* = 0.009, Cramer’s V = 0.20). “How healthy are you compared to your same age peers?”

We also examined the relationship between health and sports as sports can increase quality of and satisfaction with life. Respondents were instructed to respond to a 10-point Likert scale where 1 denoted ‘I feel absolutely unhealthy compared to my age group’ and 10 meant ‘I feel absolutely healthy compared to my age group’. The relationship between sport activity and health as measured by a subjective self-report was significant but relatively weak (Cramer’s V = 0.2; *p* = 0.009). Those actively engaged in sporting responded by scoring 7.0 or above in greater proportions (37.8%) than those who already quit (26.5%) or never actively did any sports (22.3%). Inversely, those actively sporting scored 4.0 or less in the smallest numbers (13.3%) on personal health assessment whereas those who quit scored 4.0 and less in 20.4% and those who never did sports in 33.2% (Fig. 2.). In essence, elderly who maintained regular physical activity reported much better personal health than those who stopped or never did any sports at all.

**Fig. 3:**
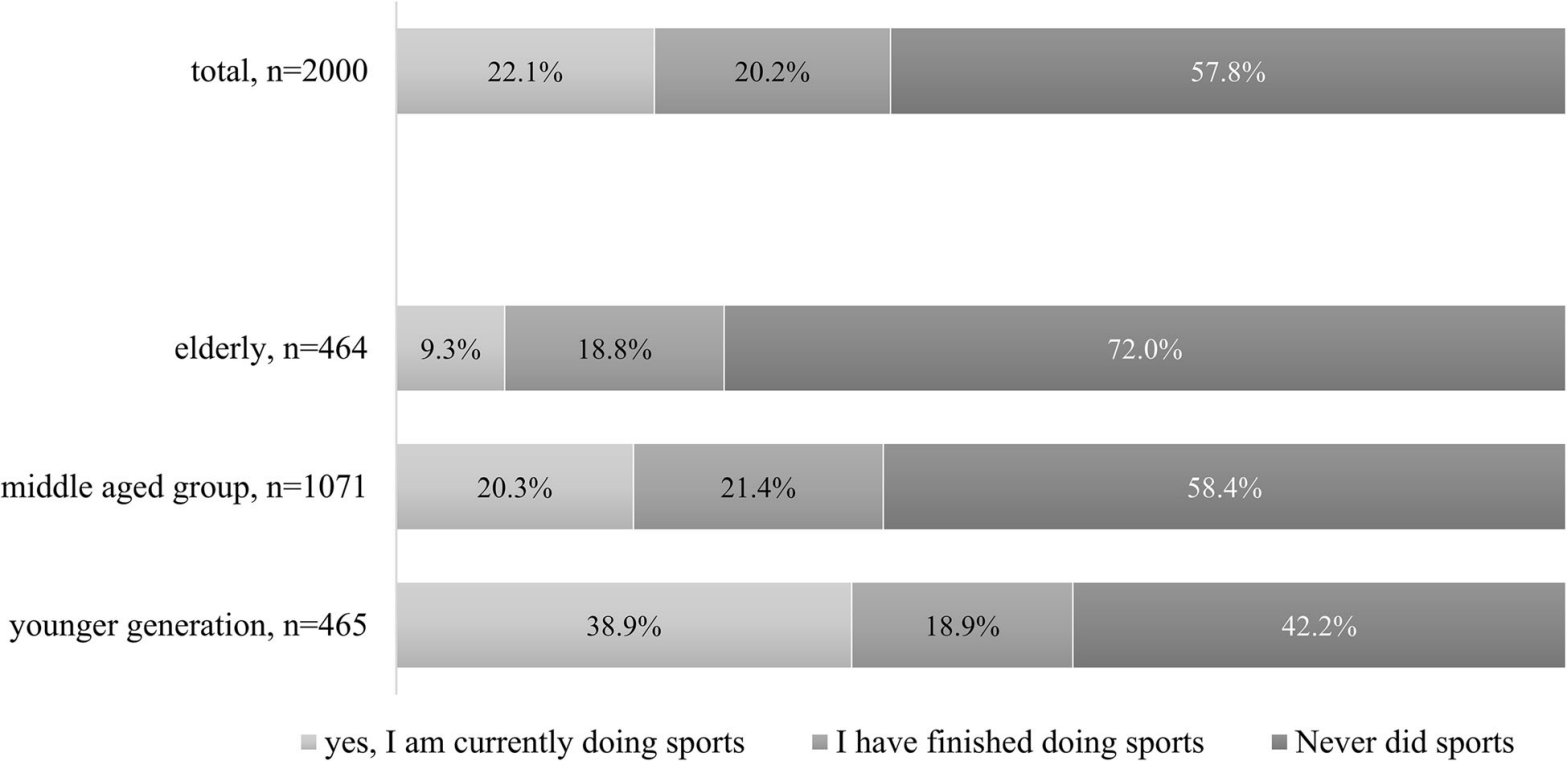
Frequency of sporting (p ‹ 0,001, Cramer’s V = 0,18). “Do you frequently do sports?”

In order to evaluate the second hypothesis, we explored the proportions of those sporting and reasons for not exercising. Figure 3 shows that 22.1% of the total sample was actually engaged in some form of physical activity. An additional 20.2% was formerly active but stopped working out completely. From a generational perspective, only 9.3% of elderly did regular physical exercise compared to 20.3% in the middle aged and 38.9% in the younger generations. What is interesting to note was that the smaller number of elderly exercising was not the consequence of old age but because 72.0% of this generation initially never did any sports before retirement. To understand why people refrain from physical exercise, we asked respondents to select multiple choices from a list of reasons.

**Fig. 4:**
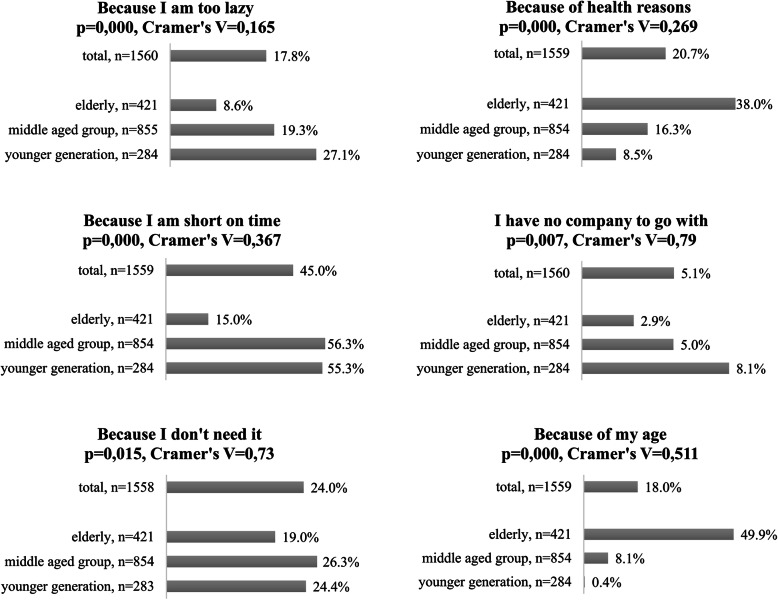
Reason for not sporting: „Why don’t you currently engage in any sports?”

Figure 4 shows that the most frequently cited reason was lack of time. This however was mainly a characteristic of the younger and middle-aged generations (55.3 and 56.3% respectively). Those in the older generation cited lack of time only 15%. Their main causes for non-sporting included ‘health reasons’ (38%) and ‘age’ (49.9%). About 19% said they did not do sports because they ‘did not feel the need of it’ which was less than reported by the other two generations. When checking for non-sporting reasons in the elderly, we found that 50.9% suffered some form of disease that may have been behind reduced activity. The same ratio was 17.9% for middle-aged and 4.7% for younger populations. We also checked for ‘age’ as a reason for no sports in our older group. Age a cause was cross checked against the item ‘Sport is for younger people’ filtered for the 60-above sample. Results revealed a positive but not very strong relationship between the two items (Cramer’s V = 0.16; *p* = 0.024). That is, non-sporting in the elderly may be explained by health reasons that prevent them from actively exercising but also by cultural thinking suggesting that beyond a certain age working out is not socially preferable.

**Fig. 5:**
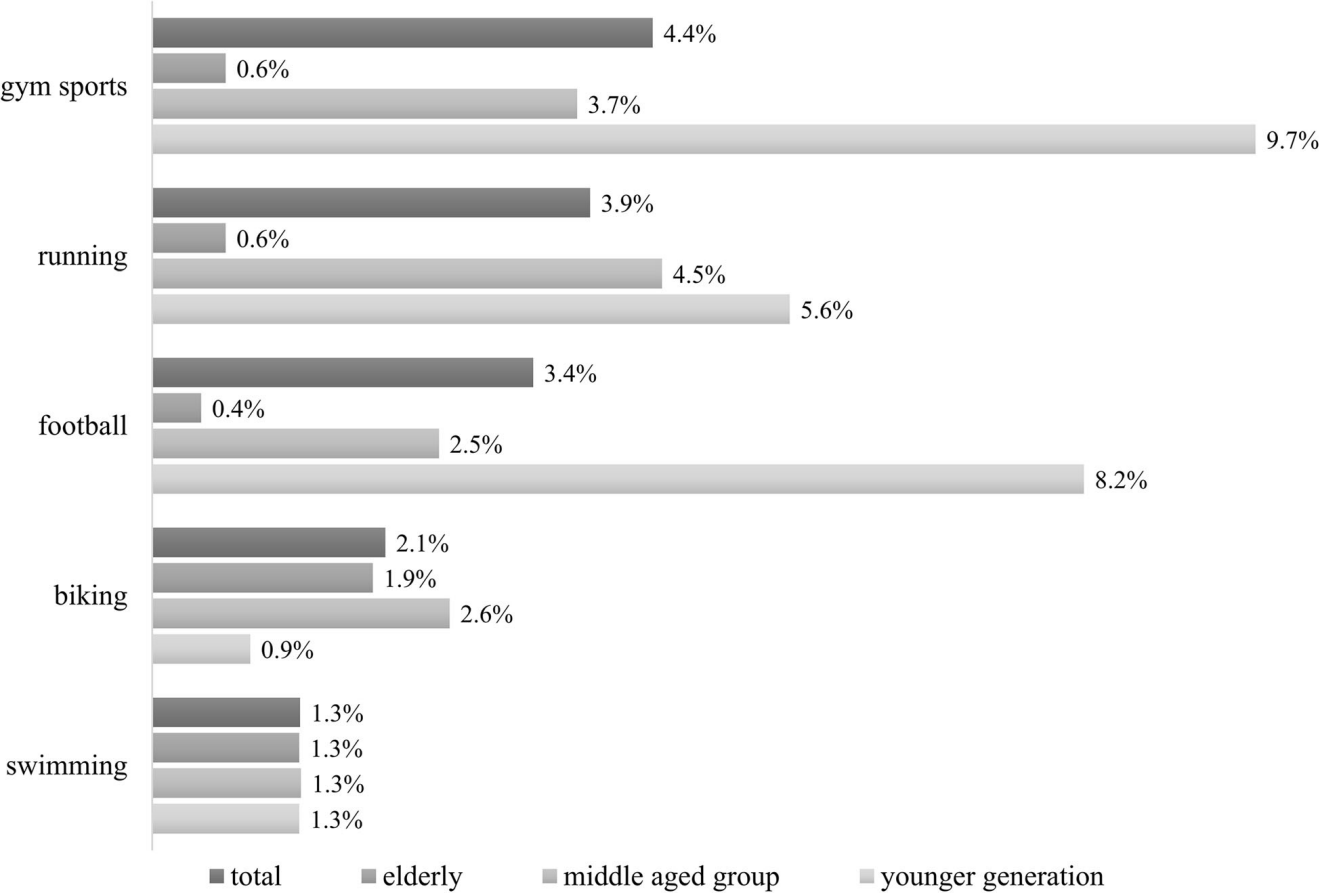
Proportion of sport activities in relation to all responses (p ‹ 0.001, Cramer’s V = 0,46). “What sports are you primarily engaged in?”

To evaluate the third hypothesis, different sport activities of the elderly were examined. The five most frequently reported sport activities were used to cross check across generations (Fig. 5). Activities and age groups showed a relatively strong relationship (Cramer’s V = 0.46; *p* ‹ 0.001). Older generations are not engaged in running, football and gym sports but more into biking and swimming instead. They outperform younger generations (1.9% vs 0.9%) and are close (1.9% vs 2.9%) to the middle-aged group in biking. Swimming is the only cross-cutting sport that an equal number of peers enjoyed.

**Fig. 6:**
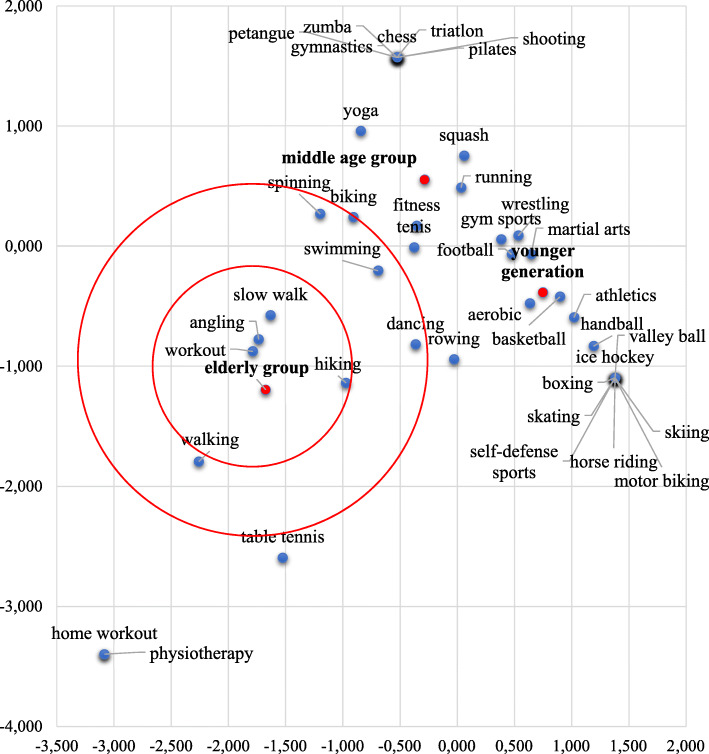
Correspondence map of sport activities (*p* ‹ 0.001, Inertia = 0.42). “What sports are you primarily engaged in?”

We employed a different approach as well to cluster activities by age groups that describe these groups best. The resulting correspondence analysis is reported in Fig. 6. The inertia value of the analysis between sport activity types and age groups was 0.42 which is very similar to the Cramer’s V obtained above. This map confirms that gym sports, football and running are not characteristic of the elderly, these are closer to the other two generations. They are more engaged in walking, hiking and less intense sports (including angling) whereas middle-aged generations are into fitness and biking. Swimming is the sport that is relatively equal distance from all three age groups. Some elderly also seem to enjoy spinning and dancing (these are on the edge of their clusters) however these are distant characteristics of the elderly group. Table tennis was a sport that was farthest from the middle-aged and young populations leaving this activity exclusively for the elderly.

Finally, we took a major interest in predicting self-reported health outcomes of elderly respondents by a set of explanatory variables. A hierarchical regression model was developed using items concerning value orientation, feelings concerning sports, frequency of sporting, life satisfaction, pace of living (slow and rapid) and BMI. To create the dependent variable, we averaged results achieved on items assessing general well-being, physical strength, mental health, physical activity and general fitness. Answers were provided on the usual Likert scale (1 = not at all true, 5 = very true). As for the independent variables, we also created four factors we named ‘slow’ and ‘rapid’ lifestyle. These were derived from the 45 item Lifestyles Inspirations Scale where items for a single dimension had been summed up and used as a new variable. We ended up with a variable for slow and rapid pace of living as well as with traditional and modern value orientations. We entered lifestyle variables in the 1st round of our hierarchical model, because these are assumed to have a stronger impact on the dependent variable. The 2nd round of our model contains variables which are not related to lifestyle.

To ensure that multicollinearity and homoscedasticity were not biasing the estimation process, tests had been evaluated and found that there were no issues concerning the above. We also made efforts to identify and remove outliers from the analysis, again, to make the final estimates relatively unbiased. Outliers whose values were < ± 2.0 standardized residuals had been removed from further analyses. The full and final model was significant (F = 65.651; p ‹ 0.001). Figure 6 shows the final regression model with only significant variables reported. The final model achieved an R^2^ = 0.564, that is, the current set of independent variables explained 56.4% of the variance in self-reported health of elderly respondents. In other words, an additional 43.6% of variance remained unexplained by the model suggesting that other aspects of personal health are to be measured. Beta weights (standardized coefficients) inform us about the relative ranking of each variable in relation to the dependent. Rapid lifestyle and satisfaction with life were the most important (0.400 and 0.316) followed by slow pace of life and BMI (− 0.181 and − 0.133, respectively) (Table 2.). B values however show the unit increase in the dependent variable caused by 1 unit increase in the independent. That is, a 1-point increase on the rapid pace of life item would correspondingly increase personal health by a score of 0.43. A similar 1-point increase on the life satisfaction item would increase personal health by 0.99 points. Comparing the effect of the two, increasing life satisfaction would double self-reported personal health of the elderly as opposed to increasing the speed (or the perception) of life. We also saw that weight gain (1-point increase on BMI) will decrease personal health scores by 0.18 points, confirming the relationship we reported earlier in this paper. While not the most relevant factor of the dependent variable by beta weights in our regression model, frequent engagement in sports achieves the greatest impact on self-reported personal health, thus supporting the main argument about physical activity of this paper. To measure the impact of frequent engagement in sports we used a dummy variable which was coded 1 if a respondent does sports regularly. In our final model, being engaged in regular sports would increase the personal health score by 1.91 points. Thus, we conclude that physical activity can be most effective to improve personal health of the elderly in this sample.

**Table 2:** Coefficients of the hierarchical linear regression model

	Unstandardized Coefficients		Standardized Coefficients	t	Sig.
	B	Std. Error	Beta		
(Constant)	2.345	0.292		8.023	0.000
Level 1					
Faster pace of life	0.043	0.005	0.4	9.233	0.000
Slower pace of life	0.021	0.005	−0.181	−4.19	0.000
Traditional value orientation	0.011	0.004	0.116	3.02	0.003
Level 2					
Satisfaction with life	0.099	0.013	0.316	7.546	0.000
BMI index	0.018	0.005	−0.133	−3.479	0.001
Regular exercising/sport activity	0.191	0.087	0.09	2.212	0.028

## Discussion

This paper set out to evaluate whether satisfaction with life and health are linked in the elderly. It also aimed to examine reasons behind non-participation in sports activities. Finally, we built a regression model to predict self-reported health of elderly. Considering our results, we confirmed the positive relationship between satisfaction with life and self-reported health. However, this relationship did not hold for the elderly in our sample. While it seems logical that health and life satisfaction go together, this was not reinforced in our sample. Explanation may be that the sample size was not adequate to support statistical conclusions. It may also be the case that life satisfaction is a very complex idea and was not primarily defined by good health in our elderly sample, however, this hypothesis needs further research to be justified. Conversely, the relationship between active sporting and personal health was confirmed, similar to some of previously presented research [8, 9, 14, 15]. Those who had actively been engaged in sport activities reported better personal health (37,8%) than those who had stopped exercising (26,5%) or never did any sports (22,3%). A comprehensive European study reported the overall health of elderly significantly better when they had been active in exercising (55.1%) compared to those who never exercised (11.2%). ‘Good’ overall health was reported much lower in Hungary (27.5%) in the actively exercising elderly group but was slightly higher (15.7%) in the non-exercising group [34]. There may however be a small bias in our data concerning those who had to stopped exercising due to health reasons, but the difference is still strong between our actively exercising and never exercised groups.

Among the many potential causes why elderly do not engage in sports the lack of exercising culture was one of our hypotheses to explain outcomes. We saw that only 9.3% of our elderly sample had been actively doing sports compared to 38.9% in the younger group. Nevertheless, data also revealed that the relatively small proportion of exercising elderly was not due to people leaving sports behind but because 72% of the sample originally never did any exercise at all throughout their lives. According to the 2017 Eurobarometer Survey, 25% of Hungarian elderly exercised regularly and 33% walked 5 days a week at least for 10 min [34]. The ELEF 2014 Survey estimated the proportion of actively sporting elderly far lower. The survey recorded in 2014 placed the proportion of sporting elderly at 12% [27], but if WHO recommendations were considered, proportion of exercising elderly men was 4% whereas only 3% of elderly women did sports [28]. Research done by Kith in 2017 documented 30.6% of older people doing sports once or twice weekly, only 2% said they had exercised daily. About half (51.4%) that sample reported not sporting at all. Those engaged in sports once a month reached 15.6% [62]. When we explored reasons behind non-exercising, elderly reported two major causes: health reasons (38%) and their age (49.9%). According to the Eurobarometer Survey, elderly in Hungary refused to exercise mainly due to existing health conditions (illness) in 44.5% and due to lack of motivation (25%). When we contrasted the ‘health’ related answer with the self-report of any disease that prevented full functioning, over half (50.9%) of our elderly sample reported at least one limiting disease. ‘Age’ was also a limiting not only because the presence of a disease as already discussed but also because our elderly agreed with a social opinion that ‘sport is for the younger’. In essence, we found evidence that members of the older generation may be restricted by health issues that prevents them from active exercising however our data confirmed that non-sporting in the elderly is also a factor of cultural views and values.

When we explored what sports define our elderly, not surprisingly we found that intense activities (football, running and gym workout) are not characteristic of the older generation. They preferred sports like walking, hiking and dancing. Table tennis was an activity that was exclusively characteristic of the elderly. In the research of Kith, elderly selected swimming and walking in greater numbers (16.4 and 16%, respectively) followed by hiking (8.7%) [63]. We found a strong generational difference across various sports, but most importantly saw that swimming was the sport all generations equally enjoyed. When we think of supporting the elderly to actively exercise, we have to consider giving priority and funding those sports where they are not limited by diseases and feel confident. Our correspondence analysis was a useful tool to highlight sports that are very characteristic of the elderly vs those which distinguish the middle-aged and young generations.

Finally, we attempted to predict self-reported health in our elderly sample. The set of independent variables that emerged significant were lifestyle factors (slow and rapid pace of living), traditional value orientation, satisfaction with life, BMI and frequent exercising. These variables explained 56.4% of the variance in health.

## Conclusions

Considering our results, we confirmed the positive relationship between satisfaction with life and self-reported health and relationship between active sporting and personal health. We also confirmed that rapid pace of living and satisfaction with life increased personal health, the biggest gain in health was by frequent physical activity. A unit increase in the frequency of exercising (for example an additional extra workout day per week) doubled the health benefit (increased self-reported health appraisal by a factor of 1.9) for the elderly. Therefore, the main conclusion of this paper is that physical activity can be the most effective way to improve personal health of our senior citizens.

### Limitations

Authors acknowledge that the study was based on an adequate sample size, however, in certain analyses sample size may have dropped lower than required for the specific statistics. Also, while the instrument used in this research was ensured of face validity, actual validity testing had not been performed.

## Data Availability

The datasets generated and/or analyzed during the current study are not publicly available because some results are still being analyzed but are available from the corresponding author on reasonable request.

## Abbreviations

ELEF2014: European Health Interview Survey (EHIS)
EUPASS: European Physical Activity Surveillance System
IPAQ: International Physical Activity Questionnaire
KSH: Central Statistical Office

## References

[1] United Nations, Department of Economic and Social Affairs, Population Division. World Population Prospects: The 2017 Revision, Key Findings and Advance Tables. New York: United Nations; 2017;11. https://reliefweb.int/sites/reliefweb.int/files/resources/WPP2017_KeyFindings.pdf Accessed 09 Jul 2019.

[2] Hegedüs R, Törőcsik M, Németh P, Resperger R (2018). Ageing Magyarországon - Generációs eltérések a korérzékelésben. Conference proceedings of the Demográfiai változások, változó gazdasági kihívások Nemzetközi Tudományos Konferencia, Magyarország.

[3] Központi Statisztikai Hivatal (KSH). Magyarország népességének száma nemek és életkor szerint. Interaktív korfa: 01.01. 2019. https://www.ksh.hu/interaktiv/korfak/orszag.html Accessed 09 Jul 2019.

[4] Monostori J, Őri P, Molnár ES, Spéder ZS, editors. Demográfiai portré 2009. Budapest: KSH NKI; 2009 p 79–87 https://wwwdemografiahu/kiadvanyokonline/indexphp/demografiaiportre/article/view/275/213 Accessed 09 Jul 2019.

[5] Monostori J, Őri P, Spéder Zs, editors. Demográfiai portré 2018. Budapest: KSH NKI; 2018 p 127–145 https://demografiahu/kiadvanyokonline/indexphp/demografiaiportre/article/view/2726/2639 Accessed 09 Jul 2019.

[6] European Union. The 2018 Ageing Report. Underlying Assumptions & Projection Methodologies. European Economy Institutional Paper 065. 2017. https://ec.europa.eu/info/sites/info/files/economy-finance/ip065_en.pdf Accessed 09 Jul 2019.

[7] Központi Statisztikai Hivatal (KSH). Születéskor várható átlagos élettartam, nemenként: 2003–2017. A társadalmi haladást mérő mutatószámrendszer. https://wwwkshhu/thm/2/indi2_8_1html Accessed 09 Jul 2019.

[8] Warburton DE, Bredin SSD (2017). Health benefits of physical activity: a systematic review of current systematic reviews. Curr Opin Cardiol.

[9] Lera-López F, Marco R (2018). Sports participation, physical activity, and health in the European regions. J Sports Sci.

[10] Umpierre D, Ribeiro PA, Kramer CK, Leitao CB, Zucatti AT, Azevedo MJ (2011). Physical activity advice only or structured exercise training and association with HbAIc levels in type 2 diabetes: a systematic review and meta-analysis. JAMA..

[11] Zhou XY, Yan L, Wang L, Wang J (2016). Association between physical activity and colorectal cancer risk and prognosis: a meta-analysis. Cancer Treat Res Commun.

[12] Richards J, Jiang X, Kelly P, Chau J, Bauman A, Ding D (2015). Don't worry, be happy: cross-sectional associations between physical activity and happiness in 15 European countries. BMC Public Health.

[13] Van Dyck D, Teychenne M, Sarah A, McNaughton SA, Bourdeaudhuij I, Salmon J (2015). Relationship of the Perceived Social and Physical Environment with Mental Health-Related Quality of Life in Middle-Aged and Older Adults: Mediating Effects of Physical Activity. PLoS One.

[14] Lok N, Lok S, Canbaz M (2017). The effect of physical activity on depressive symptoms and quality of life among elderly nursing home residents: randomized controlled trial. Arch Gerontol Geriatr.

[15] Mariolis A, Foscolou A, Tyrovolas S, Piscopo S, Valacchi G, Tsakounta N, Zeimbekis A, Bountziouka V, Gotsis E, Metallinos G, Tyrovola D, Tur JA, Matalas AL, Lionis C, Polychronopoulos E (2016). MEDIS study group. Successful Aging among Elders Living in the Mani Continental Region vs. Insular Areas of the Mediterranean: the MEDIS Study. Aging Dis.

[16] Apor P, Babai L (2014). A fizikai aktivitás lassítja az öregedéssel járó teljesítőképesség-romlást. Orv Hetil.

[17] Thomas E, Battaglia G, Patti A, Brusa J, Leonardi V, Palma A, Bellafiore M (2019). Physical activity programs for balance and fall prevention in elderly: A systematic review. Medicine (Baltimore).

[18] Ács P, Hécz R, Paár D, Stocker M (2011). A fittség (m)értéke. A fizikai inaktivitás nemzetgazdasági terhei Magyarországon. Közgazdasági Szemle.

[19] Varo JJ, Martínez-González MA, Irala-Estévez J, Kearney J, Bibney M, Martínez JA (2003). Distribution and determinants of sedentary lifestyles in the European Union. Int J Epidemiol.

[20] Lee IM, Shiroma EJ, Lobelo F, Puska P, Blair SN, Katzmarzyk PT. Effect of physical inactivity on major non-communicable diseases worldwide: an analysis of burden of disease and life expectancy. Lancet. 2012;380:219–229. 10.1016/S0140-6736(12)61031-9. 10.1016/S0140-6736(12)61031-9 PMC3645500 22818936

[21] Zhai L, Zhang Y, Zhang D (2014). Sedentary behaviour and the risk of depression: a meta-analysis. British Journal of sports medicine. BJSM Online First.

[22] World Health Organization. Global Strategy on Diet, Physical Activity and Health. 2010. https://www.who.int/dietphysicalactivity/publications/9789241599979/en/ Accessed 09 Jul 2019.

[23] EU Physical Activity Guidelines Recommended Policy Actions in Support of Health-Enhancing Physical Activity. 2008. https://eacea.ec.europa.eu/sites/eacea-site/files/eu-physical-activity-guidelines-2008.pdf Accessed 09 Jul 2019.

[24] US Department of Health and Human Services. Physical Activity Guidelines for Americans. 2nd edition. 2018. https://health.gov/paguidelines/second-edition/pdf/Physical_Activity_Guidelines_2nd_edition.pdf Accessed 09 Jul 2019.

[25] Hegedüs R, Törőcsik M, Németh P. Recent age-perceptions in Hungary. In conference proceedings of the 7th M-Sphere Conference 2018 Multidisciplinarity in Business & Science: 12-14 Dec 2018; Zagreb, in press.

[26] Global age-watch index. Insight report, summary and methodology. HelpAge International. 2015. http://www.helpage.org/global-agewatch/reports/global-agewatch-index-2015-insight-reportsummary-and-methodology/ Accessed 09 Jul 2019.

[27] Központi Statisztikai Hivatal (KSH). A 2014-ben végrehajtott Európai lakossági egészségfelmérés eredményei. Műhelytanulmány Budapest; 2018:31–47. http://www.ksh.hu/docs/hun/xftp/idoszaki/elef/elef2014_osszefoglalo.pdf Accessed 09 Jul 2019.

[28] Boros J. Egészség időskorban. In: Giczi J, editor. Ezüstkor: korosodás és társadalom. Budapest: KSH; 2017:35–50. http://www.ksh.hu/docs/hun/xftp/idoszaki/pdf/korosodas.pdf Accessed 09 Jul 2019.

[29] Bilotta C, Bowling A, Nicolini P, Casé A, Pina G, Rossi SV, Vergani C (2011). Older People’s quality of life (OPQOL) scores and adverse health outcomes at a one-year follow-up. A prospective cohort study on older outpatients living in the community in Italy. Health Qual Life Outcomes.

[30] Zs R. Testedzés és öregedés. Gerontológia. 2008:510–7.

[31] Apor P (2011). A cardiovascularis kockázat kapcsolata a fizikai aktivitással és a fittséggel. Orv Hetil.

[32] Pavlik G. A rendszeres edzés szerepe az egészség megőrzésében. Hipertónia Kardiovas Rendszer. 2011:14–8.

[33] Pavlik G. A rendszeres fizikai aktivitás szerepe betegségek megelőzésében, az egészség megőrzésében. Egészségtudomány. 2015;59 Suppl 2:11–26. http://egeszsegtudomany.higienikus.hu/cikk/2015-2/Pavlik.pdf Accessed 09 Jul 2019.

[34] Eurobarometer. Sport and Phisical Activity. 2017. https://ec.europa.eu/sport/news/2018/new-eurobarometer-sport-and-physical-activity_en. Accessed 9 July 2019.

[35] Molnár ES. Az időskorú népesség jellemzői és életkörülményei. Budapest: KSH; 2004. http://docplayer.hu/1549518-Az-idoskoru-nepesseg-fobb-jellemzoi-es-eletkorulmenyei.html Accessed 09 July 2019.

[36] Simon IÁ, Kajtár G, Herpainé LJ, Müller A (2018). A fizikai aktivitás és a mentális egészség jelentősége a 60 év fölötti korosztály életében. Képzés Gyakorlat.

[37] Rejeski WJ, Mihalko SL (2001). Physical activity and quality of life in older adults. J Gerontol.

[38] BZs O, Bognár J, Herpainé LJ, Kopkáné PJ, Vécseyné KM (2011). A survey of the living conditions and life quality of elderly people. Stud UBB Educ Artis Gymnast.

[39] BZs O, Bognár J, Herpainé LJ, Kopkáné PJ, Vécseyné KM. Lifestyle and living standards of elderly men in eastern Hungary. Phys Cult Sport Stud Res. 2011:69–79 10.2478/v10141-011-0016-6.

[40] Olvasztóné BZs, Herpainé LJ, Bognár J, Kopkáné PJ. Idős emberek életkörülményeinek és egészségmagatartásának vizsgálata. Egészségfejlesztés. 2011;52 Suppl 5–6:24–30. http://folyoirat.nefi.hu/index.php?journal=Egeszsegfejlesztes&page=article&op=view&path%5B%5D=121 Acessed 09 Jul 2019.

[41] Székács B (2005). Geriátria az időskor gyógyászata.

[42] Kopkáné PJ. A rendszeres fizikai aktivitás hatása az idős kori függetlenség megőrzésére 60 év fölötti nők esetében: randomizált kontrollált kísérlet. Doktori disszertáció, Budapest:Testnevelési Egyetem; 2014. http://real-phd.mtak.hu/335/2/kopkaneplachyjudit.m.pdf Acessed 09 Jul 2019.

[43] Sidó Z, Szamosi K. Az idős kor és sport. Hippocrates. 2005;5:299–302. http://hippocrateslap.hu/uploads/ujsag/2005-5/Az-idoskor-es-a-sport.pdf Acessed 09 July 2019.

[44] Tóth M. Szédülés és elesés idős korban. Osteológiai Közlemények. 2008:1:18–24. https://www.doki.net/tarsasag/mrtos/upload/mrtos/document/2008118.pdf Acessed 09 Jul 2019.

[45] Pető Z. Az időskori depressziók előfordulása, tünetei és felismerése. Hippocrates. 2004:2:125–133. http://hippocrateslap.hu/uploads/ujsag/2004-2/az-idoskori-depresszio-elofordulasa-tunetei-es-felismerese.pdf Acessed 09 Jul 2019.

[46] Juhász I, Kopkáné PJ, Kiszela K, Bíró M, Müller A, Révész L. Egri időskorúak rekreációs fizikai aktivitásának hatása a kardiorespiratorikus rendszerre. Magyar Sporttudományi Szemle. 2015;16 Suppl 3:4–8. http://mstt.hu/wp-content/uploads/MSTT-Szemle-2015_3.pdf Acessed 09 Jul 2019.

[47] Pfau SCh, Pető K, Bácsné BÉ. A fizikai aktivitás, mint egészségbefektetés. Egészségfejlesztés, 2011;60:1 http://folyoirat.nefi.hu/index.php?journal=Egeszsegfejlesztes&page=article&op=view&path%5B%5D=354 Acessed 09 Jul 2019.

[48] Lampek K, Rétsági E, editors. Egészséges idősödés. Az egészségfejlesztés lehetőségei idős korban. Pécs: Pécsi Tudományegyetem Egészségtudományi Kar; 2015. https://www.etk.pte.hu/protected/OktatasiAnyagok/%21Palyazati/sport2/EgeszsegesIdosodesJ.pdf Acessed 09 Jul 2019.

[49] Ainsworth BE (2009). How do I measure physical activity in my patients? Questionnaires and objective methods. Br J Sports Med.

[50] Acs P, Premusz V, Morvay-Sey K, Kovács A, Makai A, Elbert G (2018). A sporttal, testmozgással összefüggésben lévő mutatók változása Magyarországon és az Európai Unióban az elmúlt évek eredményeinek nyomán. Sport.

[51] Tudor-Locke C, Bassett DR (2004). How many steps/day are enough? Preliminary pedometer indices for public health. Sports Med.

[52] IPAQ Research Committee. Guidelines for Data Processing and Analysis of the International Physical Activity Questionnaire (IPAQ). 2005. https://www.academia.edu/5346814/Guidelines_for_Data_Processing_and_Analysis_of_the_International_Physical_Activity_Questionnaire_IPAQ_Short_and_Long_Forms_Contents Acessed 09 Jul 2019.

[53] Acs P, Betlehem J, Olah A, Bergier J, Melczer CS, Premusz V, Makai M. Measurement of public health benefits of physical activity: Validity and reliability study of the International Physical Activity Questionnaire in Hungary. BMC Public Health 2020, S20, in press. 10.1186/s12889-020-08477-z. 10.1186/s12889-020-08508-9 PMC7429907 32799846

[54] Cleland CL, Hunter RF, Kee F, Cupples ME, Sallis JF, Tully MA (2014). Validity of the global physical activity questionnaire (GPAQ) in assessing levels and change in moderate-vigorous physical activity and sedentary behaviour. BMC Public Health.

[55] Acs P, Betlehem J, Oláh A, Bergier B, Morvay-Sey K, Makai A, Premusz V. Cross-cultural adaptation and validation of the global physical activity questionnaire among healthy Hungarian adults. BMC Public Health 2020, S20, in press. 10.1186/s12889-020-08508-9. 10.1186/s12889-020-08477-z PMC7429946 32799854

[56] Eurobarometer. Physical Activity. 2002. http://ec.europa.eu/commfrontoffice/publicopinion/archives/ebs/ebs_183_6_en.pdf Accessed 09 Jul 2019.

[57] Eurobarometer. Sport and Physical Activity. 2010. http://ec.europa.eu/commfrontoffice/publicopinion/archives/ebs/ebs_334_en.pdf Accessed 09 Jul 2019.

[58] Eurobarometer. Sport and Physical Activity. 2013. https://ec.europa.eu/commfrontoffice/publicopinion/archives/ebs/ebs_412_en.pdf Accessed 09 Jul 2019.

[59] Törőcsik M, Szűcs K, Nagy Á, Lázár E (2019). Életstíluscsoportok Magyarországon a digitalizáció korában. Replika.

[60] Smith W, Clurman A (1997). Rocking the ages.

[61] Törőcsik M (2011). Fogyasztói magatartás - insigh, trendek, vásárlók.

[62] Törőcsik M, Kehl D, Szűcs K. Generációs gondolkodás – A Z és az Y generáció életstíluscsoportjai. Market Menedzsment. 2014;48 Suppl 2 special issue:3–15. https://journals.lib.pte.hu/index.php/mm/article/view/861 Accessed 09 Jul 2019.

[63] Kith N. Sport és versenyképesség Doktori értekezés. 2018 https://dea.lib.unideb.hu/dea/bitstream/handle/2437/242364/PhD_dolgozat_Kith_Nikoletta_20170623__1_.pdf?sequence=1&isAllowed=y Accessed 13 Jul 2019.

